# Influence of Afforestation on the Species Diversity of the Soil Seed Bank and Understory Vegetation in the Hill-Gullied Loess Plateau, China

**DOI:** 10.3390/ijerph14101285

**Published:** 2017-10-24

**Authors:** Ning Wang, Juying Jiao, Yanfeng Jia, Dongli Wang

**Affiliations:** 1 State Key Laboratory of Soil Erosion and Dryland Farming on the Loess Plateau, Institute of Soil and Water Conservation, CAS & MWR, Yangling 712100, China; nwang123456@snnu.edu.cn; 2 School of Geography and Tourism, Shaanxi Normal University, Xi’an 710119, China; 3 College of Water Conservancy, Shenyang Agriculture University, Shenyang 110866, China; yanfengjia@syau.edu.cn; 4 College of Environmental Science and Engineering, Liaoning Technical University, Fuxin 123000, China; wangdongli@lntu.edu.cn

**Keywords:** afforestation, soil erosion, species diversity, soil seed bank, abandoned land

## Abstract

The Chinese Loess Plateau region has long been suffering from serious soil erosion. Thus, large-scale afforestation has continued during the past decades in order to control soil erosion. Afforestation can dramatically alter nutrient cycles, affect soil-carbon storage, and change hydrology. However, it is unknown how afforestation influences species diversity of the soil seed bank and understory vegetation compared with spontaneous restoration of abandoned land. Forest land with trees planted 30 years ago, abandoned slope land restored spontaneously for 30 years, and the corresponding slopes with remnant natural vegetation were selected as sampling sites. The species richness both in the soil seed bank and vegetation was significantly higher on the afforested slope compared to the spontaneously restored abandoned land. The species similarity between the afforested slope and the remnant slope land was high both in the soil seed bank and standing vegetation compared to the abandoned land. The soil seed bank density varied from 1778 ± 187 to 3896 ± 221 seeds/m^2^, and more than half of it was constituted by annual and biennial species, with no significant difference among sampling habitats. However, the afforested slope had higher seed density of grass and shrub/subshrubs compared to the abandoned slope. The present study indicates that in the study region, characterized by serious soil erosion, afforestation can better facilitate vegetation succession compared to spontaneously restoration of abandoned slope land.

## 1. Introduction

Soil erosion is regarded as one of the global environmental problems which cause land degradation and ecosystem disequilibrium [1]. In the Chinese hill-gullied Loess Plateau region, soil erosion is frequent and serious because of the special climate, complex landform and intensive human activity [2,3]. Previously, a large part of the natural vegetation had been destroyed and the land turned into farmland, thus aggravating soil erosion and ecological degradation [3]. It is estimated that nearly 1.6 billion tons of soil are lost from the plateau each year, and droughts, floods, and dust storms are common occurrences [4]. Therefore soil and water conservation is a vital problem that compromises the safety of both ecosystem and society. Revegetation is an effective approach to control soil erosion and improve the ecosystem. The presence of grass or trees can reduce runoff and conserve soil and water [5,6,7]. Thus, slope farmlands have been increasingly abandoned for revegetation [2,3]. However, the spontaneous revegetation process is slow because serious soil erosion causes loss of soil nutrient and water [8], as well as of plant diaspore [9,10,11]. Seedling survival and growth are further limited by abiotic factors, such as high temperature and radiation, limited availability of soil water [12], and physic damage caused by storms and slope runoff [13,14]. Under such conditions, native species regeneration may benefit from habitat amelioration by afforestation, which counteracts the above abiotic stress factors [15,16]. To this aim, some fast-growing and drought-resisting woody species, such as *Robinia pseudoacacia* and *Caragana korshinskii*, are planted to accelerate revegetation and ecosystem restoration.

Afforestation is considered an important approach to rehabilitate degraded lands and their ecosystems [17,18]. Generally, a forest is planted to control soil erosion, improve soil traits, provide habitat for species conservation, and offer other ecological services [15,19,20]. In the artificial forest or shrubland, microclimate and nutrient cycle are modified [15,21,22] to influence the rate and trajectory of understory vegetation recovery and succession [23,24,25]. Herbaceous understory plays a key role in the restoration of biodiversity and in the conservation of soil and water in plantations. In temperate broadleaved forests, it forms the richest stratum in terms of plant diversity, and it influences the healthy development of the whole forest [26]. However, since afforestation influences the habitat and affects the understory plant recruitment and growth, intense debate surrounds the extent to which these anthropogenic forests protect or degrade biodiversity [24,27,28]. Tree species selections and site environments can also influence the subsequent species invasion and establishment, and later, the community structure and ecological function [19,27,29,30]. 

The presence of a native seed source in the vicinity and its successful dispersal to the artificial forest are necessary for native seeds to contribute to the restoration of native biodiversity [31]. The seed dispersal and soil seed bank both play important roles in species recruitment and succession [32,33]. Studies indicate that tree plantations can facilitate native species regeneration by attracting seed dispersal agents from nearby native communities [34]. Simultaneously, the soil seed bank is an important component of vegetation dynamics [35]. In forests, soil seed banks bear the marks of former land use and forest management, and play an important role in vegetation dynamics [36]. The historical land use is a key factor that can influence the soil seed bank composition for a long-period [37,38,39]. Although the original vegetation is destroyed during land preparation and tree planting, many species persist in the soil as seeds [37,40]. In particular, pioneer species with a large soil seed bank would control the herbaceous layer at an early stage of afforestation or after disturbance [39]. However, during the development of the plantation, many of the open-field species which are shade-intolerable will disappear and will be replaced by successional species or native understory species [41]. 

The Loess Plateau region in northwest China is well-known of its serious soil erosion. Afforestation is broadly used to control soil erosion, accelerate vegetation restoration, and improve ecological environments [18,42]. The ecological impact of afforestation becomes the focus of intense debate during the afforestation process [42,43]. Being afforestation a primary measure of intervention, its influence on the soil characteristics such as soil erodibility, soil water, soil fertility [44,45], and its ecological function on erosion control, catchment-water yield and soil carbon sequestration were the focus of previous studies [46,47,48]. However, there is scarce knowledge about whether afforestation modifies the habitat to affect structure and species diversity of the soil seed bank and understory vegetation. The objectives of the present study were to assess the influence of afforestation on the soil seed bank and understory vegetation after 30 years of natural succession.

## 2. Materials and Methods

### 2.1. Study Site

This study was carried out in Zhifanggou watershed, which is located in An’sai county, in the Loess Plateau region (109°19′30′′ E, 36°51′30′′ N) at 1010–1431 m above sea level. The watershed has a semiarid climate with an average annual precipitation of 505 mm (1970–2006). Over 60% of the precipitation falls during the rainy season (July–September), usually in the form of storms. The annual evaporation is over 1460 mm, and the mean temperature is approximately 8.8 °C (from −11 °C to 30 °C). Within the study region, the landscape includes inter-gully slopes and gully slopes, and the land surface is fragmented by deeply incised and densely distributed gullies (gully density 8.06 km·km^−2^). 

Although this area is located in the forest-steppe region, according to the literature the main body of the Loess Plateau appears to be covered by herbaceous or shrub species, but not by forest [49,50]. In fact, the native forest has almost been destroyed and replaced by typical steppe as a consequence of long-term human activity. Some native forest woody species are scattered over the steppe as isolated trees. The main native species in the different successional stages and landscapes are herbs, such as *Artemisia scoparia*, *Artemisia gmelinii*, *Artemisia giraldii*, *Lespedeza davurica*, *Stipa bungeana*, *Bothriochloa ischaemun* [44], and a few native shrubs, such as *Rosa xanthina*, *Sophora viciifolia*, and *Syringa julianae*. Broad planting species, such as *R. pseudoacacia* and *C. korshinskii* are also scattered throughout the landscapes.

### 2.2. Soil Samples and Standing Vegetation Investigation

In order to investigate the influence of afforestation on the soil seed bank, a south-facing slope planted with *R. pseudoacacia* and subject to natural succession for 30 years (Ps), and a north-facing slope planted with *C. korshinskii* and subject to natural succession for 30 years (Pn) were selected to collect soil samples. At the same time, south-facing and north-facing slope lands with remnant natural vegetation were selected (Rs and Rn, respectively). The soil samples prepared for germination experiments were collected in the four types of habitats. Nine plots, each 5 m × 5 m, were located on each habitat. At each plot, 20 soil cores (diameter 4.8 cm) were collected in the 0–10 cm soil layers. The soil samples were collected in April, July and October in 2009. Additionally, the data about two abandoned slope lands (one a south-facing slope and the other a north-facing slope) with spontaneous restoration for 30 years (As and An, respectively) were used for comparison with the afforestation site. The soil seed bank samples in these two slopes were collected in April, August, and October in 2005. Every time, six 10 cm × 10 cm soil samples of 0–10 cm in depth were collected separately from each slope. The understory vegetation and the standing vegetation on the remnant slope land and on the abandoned slope land were investigated in July of the study year. Three 1 m × 1 m quadrats were surveyed in each sampling plot. The species composition was studied by recording the species that grew in the quadrat. The density of a given species was calculated as the number of individuals of that species per square meter. The coverage of each plant species was estimated by two experts at the same time.

### 2.3. Germination Experiments

The soil seed bank was identified using the germination method after the soil samples had been concentrated. The air-dried soil samples were sieved using a pore size of 0.15 mm, and the soil retained in the sifter was the concentrated soil samples [51]. The germination experiment was conducted in a greenhouse. The concentrated soil samples were distributed over a 2 cm deep perlite layer in 24 × 15 × 5 cm plastic trays, to obtain a 0.5 cm high layer. During the experiments, the temperature in the greenhouse fluctuated between 11 and 35 °C, with a mean value of 25 °C. The germination trays were watered regularly. The seedlings were identified and removed, or replanted for later identification. The soil was dried and thoroughly stirred for the next germination period when no seedlings emerged within two weeks after the peak of seedling emergence. The germination experiment was terminated when there was no seedling emergence for four weeks, and the germination continued for approximately four months (15 March to 15 July in 2009). Also, the soil seed bank samples collected in 2005 were identified using the germination method [52].

### 2.4. Statistical Analysis

We characterized species richness, diversity and evenness in each habitat, in order to reveal the influence of afforestation on the soil seed bank and understory vegetation. Three species diversity indices were employed:

(1) Species number as the richness index (*S*).
(2) Shannon–Wiener diversity index (*H’*):
  $$H\prime =-\sum (P_{i})\ln(P_{i}),$$
(3) Pielou evenness index (*J*):
  $$J=\frac{H\prime }{\ln S},$$

where *S* is the number of species, *P_i_* is the proportion of individuals or the abundance of the *i*th species expressed as a proportion of the total number of species in the community, and ln is the natural logarithm.

In order to reveal the differences in species composition between the soil seed bank and standing vegetation in the same and different habitats, Sorensen similarity coefficient was employed:

$$CC=\frac{2w}{a+b},$$

where *CC* is the Sorensen similarity coefficient; *w* is the number of species present in both plots; *a* and *b* are the number of species in each of the investigated plot. 

Species were ascribed to functional groups: annuals and biennials, perennial grasses, perennial herbs, semi-scrubs and shrubs, trees. 

The differences in species diversity in both the soil seed bank and standing vegetation over different habitats were analyzed using ANOVA. The soil seed density was transformed using log(*x* + 1) to satisfy the homogeneity of variance assumption.

## 3. Results

**Figure 1:**
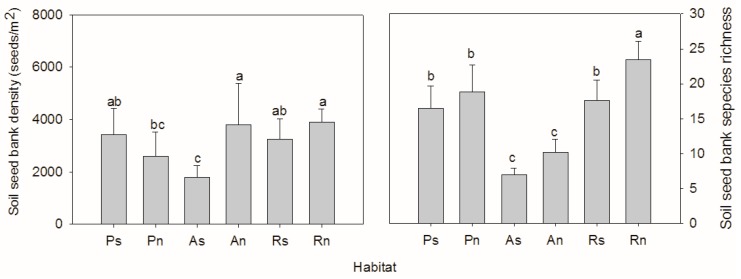
Soil seed bank density and species richness varied among different habitats (Ps/Pn—south/north-facing afforested slope land, As/An—south/north-facing abandoned slope land with spontaneous restoration, Rs/Rn—south/north-facing slope lands with remnant natural vegetation. The letters “a, b, c” above the bar indicates significant difference at the 0.05 level).

The soil seed bank density was significantly different among the habitats (F = 3.988, *p* = 0.007) (Figure 1). On the south-facing slope, the seed bank density was significantly lower on the abandoned land (1778 ± 187 seeds/m^2^) than on both the planted slope land (3415 ± 415 seeds/m^2^) and the land with remnant vegetation (3249 ± 348 seeds/m^2^), but there was no significant difference between the last two. On the north facing-slope, the seed bank density on the planted slope land (2600 ± 378 seeds/m^2^) was significantly lower than in the other two habitats. The soil seed bank species richness varied between 7.0 ± 0.4 and 23.4 ± 1.2, and was significantly different among the habitats (F = 27.235, *p* < 0.001). The species richness was significantly higher on the planted slope than on both the south- and north-facing slopes of the abandoned land. However, the species richness was higher on the slope with remnant vegetation than on the planted slope, especially on the north-facing slope. 

**Figure 2:**
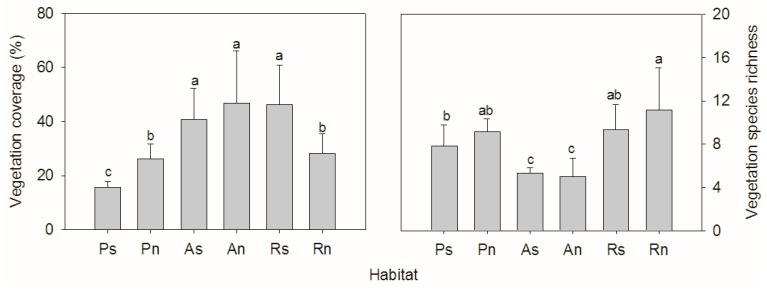
Vegetation coverage and species richness varied among different habitats (Ps/Pn—south/north-facing afforested slope land, As/An—south/north-facing abandoned slope land with spontaneous restoration, Rs/Rn—south/north-facing slope lands with remnant natural vegetation. The letters “a, b, c” above the bar indicates significant difference at the 0.05 level).

The vegetation coverage varied significantly in the different habitats (F = 10.488, *p* < 0.001) (Figure 2). The understory vegetation on the south-facing planted slope had the lowest coverage (15.8 ± 0.9%), which was significantly lower than in the other two habitats. On the north-facing slope, the understory vegetation had the lowest coverage (26.0 ± 2.4%), which was close to the vegetation coverage on the remnant slope and significantly lower than the vegetation coverage on the abandoned slope. The species richness varied between 5.0 ± 0.7 and 11.2 ± 1.6, and was significantly different among habitats (F = 9.660, *p* < 0.001). The species richness on the abandoned slope was significantly lower than on the planted slope and the remnant slope, on both south- and north-facing aspects.

In total, this study identified 88 species belonging to 35 families in the soil seed bank and in the standing vegetation (Table 1). In particular, 63 species belonging to 24 families and 50 species belonging to 20 families were identified in the soil seed bank and standing vegetation, respectively. The species belonging to composite, gramineae and leguminosae represented a large part of the identified species. The total number of species in the soil seed bank varied from 14 to 43 in different habitats. The slope remnant vegetation had the highest species number, whereas the abandoned slope land had the lowest species number. In the standing vegetation, the total species number varied from 12 to 27, and the lowest species number was recorded on the abandoned land. 

**Figure 3:**
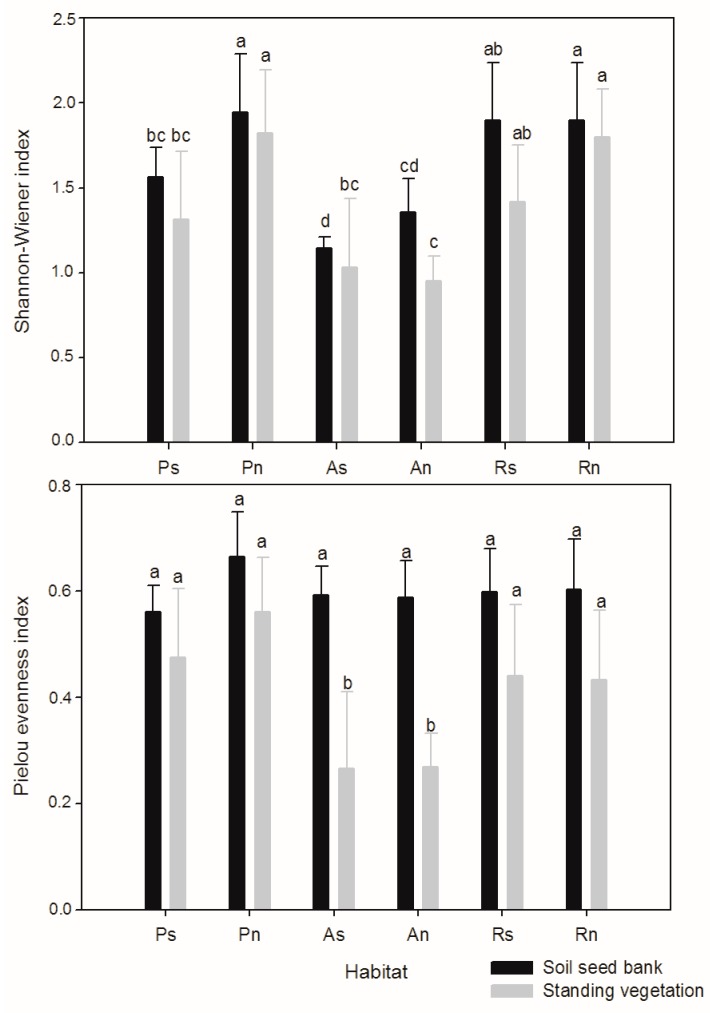
Species diversity of the soil seed bank and standing vegetation in different habitats (Ps/Pn—south/north-facing afforested slope land, As/An—south/north-facing abandoned slope land with spontaneous restoration, Rs/Rn—south/north-facing slope lands with remnant natural vegetation. The letters “a, b, c” above the bar indicates significant difference at the 0.05 level).

The species diversity indices of standing vegetation and the soil seed bank changed in the different habitats (Figure 3). The Shannon-Wiener diversity index for the soil seed bank (F = 8.887, *p* < 0.001) and standing vegetation (F = 6.101, *p* < 0.001) was significantly difference in the habitats. The abandoned slope had the lowest Shannon-Wiener diversity index compared to the planted slope and the remnant slope. However, the Pielou evenness index for the soil seed bank and standing vegetation was not significantly difference in different habitats. 

**Figure 4:**
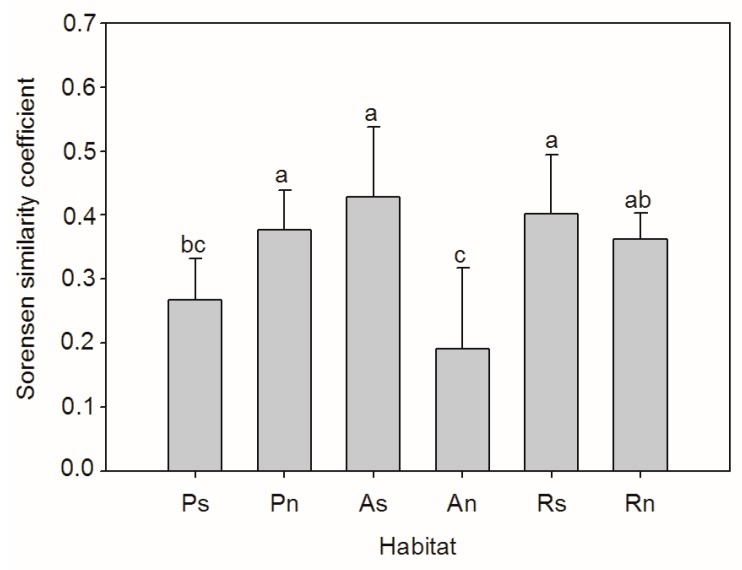
The Sorensen similarity coefficient between the soil seed bank and standing vegetation in different habitats (Ps/Pn—south/north-facing afforested slope land, As/An—south/north-facing abandoned slope land with spontaneous restoration, Rs/Rn—south/north-facing slope lands with remnant natural vegetation. The letters “a, b, c” above the bar indicates significant difference at the 0.05 level).

The Sorensen similarity coefficient between standing vegetation and the soil seed bank changed from 0.19 ± 0.05 to 0.43 ± 0.05, and was significantly difference in the different habitats (F = 6.087, *p* = 0.001) (Figure 4). On the south-facing slope, the similarity coefficient was the lowest on the planted slope, and the highest on the abandoned slope. On the north-facing slope, the similarity coefficient was the lowest on the abandoned slope, and whereas it had similar value in the other two habitats. Regarding the soil seed bank, the similarity coefficient between the planted slope and remnant slope was 0.69 and 0.70, on both the south- and north-facing slopes (for the abandoned slope and remnant slope, it was 0.55 and 0.53 on both the south- and north-facing slopes). Regarding the standing vegetation, the similarity coefficient between the planted slope and remnant slope was 0.41 and 0.52 on both the south- and north-facing slope (for the abandoned slope and remnant slope, it was 0.47 and 0.38 on both the south- and north-facing slope).

The proportion of species belonging to different function groups in the soil seed bank and standing vegetation varied among the habitats (Table 2). In every habitat, the annuals/biennials species constituted more than half of the soil seed bank density (with the exception of the south-facing remnant slope), with the species number taking more than 30%. However, in the vegetation, these species had low coverage, and even no coverage in the south-facing abandoned slope and remnant slope. The species richness of perennial herbs was more than 25% both in the soil seed bank and standing vegetation, but their soil seed bank density was low. A few species belonging to shrub and subshrub were found in the soil seed bank, but in the vegetation they had higher coverage, especially on the south-facing remnant slope and north-facing abandoned slope. In the sampling plots, the native tree species were rare both in the soil seed bank and standing vegetation.

## 4. Discussion

Significant differences in the densities and species diversity of seeds in the soil seed bank among study habitats were found in this study. The density of the soil seed bank in the plantation changed from 2600 ± 378 to 3415 ± 415 seeds/m^2^ in the 0–10 cm soil layer. This value was relatively low compared with the correspondent one determined for the abandoned slope land that underwent natural recovery for about 15 years and had a soil seed bank density greater than 10,000 seeds/m^2^ [52]. The seed bank density in the plantation was similar to the one for a plantation in the rocky mountain region of Beijing, which was also planted with *R. pseudoacacia* and other deciduous broadleaf tree or shrub species [53]. The species number of the soil seed bank ranged from 14 to 43. After 30 years of succession, the abandoned slope land had a lower species richness compared to the planted slope, on both south- and north-facing slopes. In addition, the species richness was similar on the planted slope and on the remnant slope. Moreover, the species richness in the standing vegetation was considerably higher on the planted slope than on the abandoned slope, on both the south- and north-facing slopes.

The Shannon-Wiener diversity index and Pielou evenness index of the soil seed bank and standing vegetation were not significantly different for plantation and remnant slope, but they were both higher compared to the abandoned slope land with natural restoration. Species diversity is the main indicator of community changes in the restoration of abandoned land in the Loess Plateau [54]. Therefore, these results indicated that, with respect to the abandoned land with natural restoration, the planting treatment had a positive influence on the diversity of soil seed bank and standing vegetation.

Previous studies in this region also indicated that afforestation can improve soil nutrients, vegetation structure, and species diversity [28,43,55,56]; in addition, plantations could improve available soil nutrients more significantly than natural grasslands in the gully areas where soil erosion is more serious [56]. In the *R. pseudoacacia* plantation, soil nutrients and moisture at the superficial soil layer improved with plantation succession age [57]. The planted forest and shrub land can also improve microenvironment factors. They effectively reduce sun radiation, air temperature, and soil temperature fluctuation, and enhance air humidity. Thus, in the study region with abiotic stress for vegetation regeneration, the plantation has a potentially positive role in the restoration of native biodiversity. The benefits of afforestation on the understory microenvironments and its positive role in native species succession were also found in other studies in stressed environments [15,58].

The relationship between the soil seed bank and standing vegetation changed with vegetation types, succession period, and management regime. In this study, seeds belonging to annuals and biennials represented a large part of the soil seed bank in each habitat. In contrast, in the vegetation, these species were always present in a small proportion (Table 2). This is similar to what was found in studies on the abandoned slope land in this region, which showed that the annuals and biennials can persisted in the soil seed bank with a high density [51]. Even after 30 years of succession the annuals and biennials in the standing vegetation are still more diffused in the plantation than in the remnant slope land. The seeds of grasses, perennial herbs, shrub and subshrubs had a relatively low density in the seed bank, but they were rich in species composition as a consequence of their short longevity in the seed bank. Because of their longevity and ability of vegetative regeneration, these species were the most represented in the standing vegetation [51].

In the soil seed bank, species similarity between plantation and remnant slope land was high on both the south- and north-facing slopes (similarity coefficients were 0.74 and 0.73, respectively). The dominant species in different successional stages on the abandoned slope land were investigated both in south- facing and north-facing plantation. Among these were the pioneer species *A. scoparia*, and the later stage species *S. bungeana*, *L. davurica*, *B. ischcemum*, *C. chinensis*, *A. giraldii*, *A. gmelinii*, etc. [44]. At the same time, in standing vegetation, the species similarity between plantation and remnant slope land was also higher compared to the species similarity between plantation and abandoned slope land. Many of the native perennial herbs, grasses and shrubs existed in plantations. In the study region, because of the high gully density, landscape mosaics are formed by patches of remnant vegetation, abandoned farmland, and plantation. This landscape is important for seed transmission from adjacent remnant vegetation patches to the plantation, and maintains the potential for recovery of the transformed sites [59]. Furthermore, plantation can improve the plant growth habitat, and more native species can resettle in plantations, so that the species similarity between plantation and remnant slope land is higher. 

The tree species were rare both in the soil seed bank and in the standing vegetation. Even for planted species, the soil seed bank density was low for *R. pseudoacacia*, and no seed of *C. korshinskii* was found in the seed bank. The low seed density of *R. pseudoacacia* may be due to predation in the canopy seed bank and post-dispersal. The seedling of *R. pseudoacacia* was also rare in the forest. *C. korshinskii* is a sandy shrub, and seeds of *C. korshinskii* germinate quickly after dispersal. However, on the Loess land, the seed can-not get into the soil, so nearly all the germinated seeds died because of drought. For these reasons, it was difficulty for these two planted species to regenerate from seeds in the study region. The native tree species in the study region, such as *Pyrus betulifolia*, *Salix matsudana*, *Populus simonii*, *Platycladus orientalis*, etc. spread sparsely in the background of steppe and shrub [49]. The present vegetation type and pollen records from various topographic units show that herbs were dominant in this region [60]. One previous study indicated that the development of forests on abandoned fields takes 40–50 years in the Loess area of China [54]. However, in the present study, after 30 years of succession, there is no indication that the native forest species will recolonize the region. The lack of available propagule is one critical factor that limits native forest species regeneration [31,58]. In addition, the climate and Loess soil also limit forest development in this region [49,60,61].

## 5. Conclusions

The present study indicated that there is a positive influence of afforestation on the native species restoration, with respect to species diversity of the soil seed bank and standing vegetation. After 30 years of succession, the understory species composition was more similar to the one in the remnant slope than the one in abandoned land with natural restoration. Thus, in a stressed environment with serious erosion soil, afforestation with appropriate species can help the native species to resettle. However, there was rare native shrub resettling, and no tree species resettling in the plantation. More research is needed to determine whether this is caused by a lack of available propagule, or by the climate and deep Loess soil.

## Figures and Tables

**Table 1:** The characteristics of soil seed bank and vegetation in different habitats.

Species	Soil Seed Bank (seeds/m^2^)						Vegetation Cover (%)					
	Ps	Pn	As	An	Rs	Rn	Ps	Pn	As	An	Rs	Rn
Annuals/biennials												
*Amaranthus tricolor*	2											
*Androsace septentrionalis*		23		28	4							
*Artemisia hedinii*	94	6		250	553	11	0.2					
*Artemisia scoparia*	1701	966	983	2072	1463	1448		0.1				0.1
*Bidens pilosa*	3			6								
*Bothriospermum chinense*	5	31			4	4						
*Chenopodium serotinum*			11	11	4	2						
*Digitaria sanguinalis*					2							
*Dracocephalum moldavica*	20	18	28	11	7	4	0.0			0.8		0.1
*Echinochloa crusgali*						2						
*Euphorbia humifusa*	2	15	6	67	6	13						
*Eragrostis pilosa*	17	2		6	15							
*Galium aparine*							0.3					
*Incarvillea sinensis*							0.1					
*Ixeris polycephala*		2		11		11						
*Kochia scoparia*	3				2							
*Leonurus artemisia*						17						
*Linum stelleroides*		12			15	26						
*Plantago asiatica*	2				6	2						
*Salsola collina*	61	57			2		0.6	0.9				
*Saussurea japonica*							0.1	0.1		0.2		
*Setaria viridis*	78	21	22	122	35	83				0.2		
*Sonchus oleraceus*					2							
*Swertia bimaculata*	123	370				133						
*Torilis scabra*					2							
*Torularia humilis*			11		158	50						
Perennial herbs												
*Artemisia giraldii*		12	6		2	41		0.6	0.8		9.4	1.2
*Artemisia mongolica*							0.3	0.4				0.1
*Astragalus adsurgens*		3		39	2	2						
*Astragalus scaberrimus*												0.1
*Bupleurum yinchowense*		11						0.4				
*Dendranthema indicum*		3				11				1.7		4.9
*Diarthron linifolium*								0.0				
*Geranium wilfordii*	3											
*Gerbera anandria*						7						
*Glycyrrhiza uralensis*							0.6	0.8	0.8			0.2
*Heteropappus altaicus*	3	18	6	78	20	20	0.5	0.7	0.2		0.1	
*Ixeridium chinense*	57	11	11	11	55	13						
*Ixeris sonchifolia* Bge. Hance	149	48		22	37	175	0.1					0.1
*Leontopodium leontopodioides*		2										0.3
*Linum perenne*	2											
*Melilotus officinalis*	3	17			4	18		0.1	1.7	0.7	0.1	0.3
*Oxytropis discolor*						2					0.1	
*Patrinia scabiosaefolia*	12	23		6	9	74		2.1		0.8		3.6
*Polygala tenuifolia*	17	2			11	2	0.1				0.2	
*Potentilla bifurca*								0.7				0.1
*Potentilla tanacetifolia*		66	44		2	4		0.2	0.8			0.0
*Pulsatilla chinensis*								0.2				
*Rubia cordifolia*						2						
*Serratula centauroides*									0.2			
*Thalictrum aquilegifolium*										0.7		
*Vicia amoena* Fisch. ex DC.		2								0.3		4.4
*Viola dissecta*	2	29			9	15		0.1				
*Viola philippica*							0.1					
Perennial grasses												
*Bothriochloa ischcemum*	3	11	556		103	4	0.2		29.0		1.3	
*Carex lanceolata*						20						
*Cleistogenes caespitosa*									0.2			
*Cleistogenes chinensis*	6	8			311	17	0.3	0.4			3.6	0.1
*Cleistogenes hancei*						6						0.3
*Cleistogenes squarrosa*				17					1.0	1.3		
*Helictotrichon schellianum*								0.1				
*Koeleria cristata*												0.5
*Leymus secalinus*						2						
*Melica radula*						98						
*Melica scabrosa*	54	9				24		0.2				0.4
*Pennisetum centrasiaticum*										0.2		
*Phragmites australis*							0.7	0.4				
*Poa sphondylodes*	63	193		907	134	1074						0.1
*Stipa bungeana*	743	89	39	17	11	48	9.0	4.1	0.5	2.3	0.6	0.6
*Roegneria kamoji*	2						0.1					0.0
Shrubs/Sub-shrubs												
*Ampelopsis glandulosa*										0.8		1.7
*Artemisia gmelinii*	52	180	11	89	41	326	1.3	11.8	2.2	30.0	7.7	8.5
*Buddleja alternifolia*	3				114	6					3.1	
*Caragana korshinskii*								1.2		3.3		
*Clematis fruticosa*					4	2					0.9	
*Lespedeza cuneata*						2						0.1
*Lespedeza davurica*	5	9	44	33	77	13	0.7	0.8	3.3		3.4	0.3
*Lespedeza floribunda*											3.9	
*Periploca sepium*		2					0.5				0.8	0.2
*Prinsepia utilis*											2.3	
*Sophora davidii*					24						7.9	
*Syringa oblata*						6						
*Rubus parvifolius*												0.1
*Ziziphus jujuba*											0.3	
Trees												
*Ailanthus giraldii*											0.7	
*Robinia pseudoacacia*	71	2										
*Ulmus pumila*										3.3		

Note: Ps/Pn—south/ north-facing afforested slope land, As/An—south/north-facing abandoned slope land with spontaneous restoration, Rs/Rn—south/north-facing slope lands with remnant natural vegetation.

**Table 2:** The soil seed bank density and vegetation coverage of different function groups vary among different habitats (A, Annuals/biennials, P, Perennial herbs, G, Perennial grasses, S, shrubs/sub-shrubs, T, Trees).

Items		Ps	Pn	As	An	Rs	Rn
Soil seed bank density (seeds/m^2^)	A	2109	1523	1061	2583	2277	1803
	P	247	246	67	156	151	385
	G	870	310	594	941	560	1291
	S	60	190	56	122	260	354
	T	71	2				
Soil seed bank species richness	A	13	12	6	10	17	14
	P	9	14	4	5	10	14
	G	6	5	2	3	4	9
	S	3	3	2	2	5	6
	T	1	1				
Vegetation cover (%)	A	1.2	1.1		1.2		0.2
	P	1.6	6.0	4.5	4.2	9.9	15.1
	G	10.3	5.3	30.7	3.8	5.5	2.1
	S	2.5	13.8	5.5	34.2	30.3	10.9
	T				3.3	0.7	
Vegetation species richness	A	6	3		3		2
	P	6	12	6	5	5	12
	G	5	5	4	3	3	7
	S	3	3	2	3	9	6
	T				1	1	

Note: Ps/Pn—south/north-facing afforested slope land, As/An—south/north-facing abandoned slope land with spontaneous restoration, Rs/Rn—south/north-facing slope lands with remnant natural vegetation.
